# Insect-based diets (house crickets and mulberry silkworm pupae): A comparison of their effects on canine gut microbiota

**DOI:** 10.14202/vetworld.2023.1627-1635

**Published:** 2023-08-17

**Authors:** Sathita Areerat, Pipatpong Chundang, Chalermpol Lekcharoensuk, Preecha Patumcharoenpol, Attawit Kovitvadhi

**Affiliations:** 1 Graduate Student in Animal Health and Biomedical Science Program, Faculty of Veterinary Medicine, Kasetsart University, Bangkok 10900, Thailand; 2 Department of Physiology, Faculty of Veterinary Medicine, Kasetsart University, Bangkok 10900, Thailand; 3 Department of Companion Animals Clinical Sciences, Faculty of Veterinary Medicine, Kasetsart University, Bangkok 10900, Thailand; 4 Interdisciplinary Graduate Program in Bioscience, Faculty of Science, Kasetsart University, Bangkok 10900, Thailand

**Keywords:** 16S ribosomal ribonucleic acid, canine, cricket, gut microbiota, insect, silkworm

## Abstract

**Background and Aim:**

The gut microbiome plays an important role in the overall health and well-being of dogs, influencing various physiological processes such as metabolism, nutrient absorption, and immune function. Edible insects are a sustainable and nutritious alternative protein source attracting increasing attention as a potential component of animal feeds, including pet food. However, little is known about the effects of insect-based diets on the gut microbiota of dogs. This study aimed to examine the fecal microbiota of dogs fed a diet that substituted common protein sources (poultry meal) with the house cricket (*Acheta domesticus* [AD]) or mulberry silkworm pupae (*Bombyx mori* pupae [BMp]) at different levels.

**Materials and Methods:**

Fifteen healthy adult mixed-breed dogs were systemically randomized and assigned into each block under a completed randomized block design into the following five experimental dietary groups: control diet, 10% AD, 20% AD, 7% BMp, or 14% BMp for 29 days. The amounts fed to the dogs were based on the daily energy requirement. Fecal samples were collected on days 14 and 29 and analyzed for bacterial community structure using 16S ribosomal ribonucleic acid gene sequencing.

**Results:**

At the phylum and genus levels, microbiota and their diversity were generally relatively similar among all treatments. The diets containing insects did not significantly alter the major phyla in the gut microbiome of dogs (p > 0.05). A few significant changes were found in the relative abundance of bacterial genera, with the levels of *Allobaculum* and *Turicibacter* being reduced in dogs fed a higher level of BMp. In contrast, only a decrease in *Turicibacter* was found in dogs fed the lower level of AD than the control diet (p < 0.05). *Corynebacterium* and *Lactobacillus* levels in the dogs fed 14% BMp were significantly increased compared with those in the control group (p < 0.05).

**Conclusion:**

These findings suggest that insect-based diets may slightly alter the gut microbiota of dogs. Further research is needed to fully understand the mechanisms by which insect-based diets influence the gut microbiota of dogs and the long-term potential health implications.

## Introduction

The number of pets, especially dogs, is growing rapidly as human lifestyles have changed. Humans and dogs have comparable environments, behaviors, and foods. With the trend of “pet humanization,” dogs are treated as family members. They are considered omnivores and always share their food resources, while gastrointestinal research has used the dog as a suitable model due to its anatomical and physiological similarities with humans [1]. Therefore, the gut microbiome of dogs has been investigated and the obtained findings have been applied to humans [2, 3].

A gut microbiome is a group of microorganisms consisting of bacteria, archaea, fungi, viruses, and others, living and colonizing the gastrointestinal tract of humans and animals. They also play an important role in many functions relating to the host’s health, particularly development, growth performance, digestion, and immune system function [4–6]. The major phyla identified in the dog microbiome are *Actinobacteria*, *Bacteroides*, *Firmicutes*, *Fusobacteria*, and *Proteobacteria* [7] in relative balance. The most abundant genera are *Bacteroides*, *Bifidobacterium*, *Clostridium*, *Dorea*, *Enterobacteriaceae*, *Fusobacterium*, *Lactobacillus*, and *Ruminococcus* [5, 8].

The composition and proportion of the gut microbiome are influenced by various factors, such as disease (e.g., inflammatory enteropathies, allergy, constipation, periodontitis and/or gingivitis, obesity, diabetes, and kidney disease), antibiotic administration, fecal microbiome transplantation, and diet. Various nutrients can affect the gut microbiome, such as macronutrients (protein, fat, carbohydrate, and fiber) and biotics (prebiotics, probiotics, and synbiotics) [6, 7]. It has been reported that nutritional interventions rapidly alter the gut microbiome [9]. In addition, variations in the proportion of nutrients fed to healthy dogs can influence their gut fecal microbiome [10, 11].

It is predicted that, in 2050, there will be greater demand for food as the world’s population increases. Edible insects have been suggested as potential replacements for other animal-based proteins for humans and animals [12]. Compared with common protein sources, insects have a higher ratio of edible components, better nutritional profile, minimal investment costs, short production cycles, environmental friendliness, and contribute to the Bio-Circular-Green Economy [13–16]. Numerous studies have shown that nutrient digestibility in dogs is not influenced by a diet containing insects such as the house cricket (*Acheta domesticus* [AD]), mulberry silkworm pupae (*Bombyx mori* pupae [BMp]), tropical house cricket (*Gryllodes sigillatus*), black soldier fly (*Hermetia illucens*), and yellow mealworm (*Tenebrio molitor*) [17, 18], and it is safe for human and dogs’ health [17–19]. However, only a few studies have been conducted on the gut microbiome in dogs fed a diet containing insects. Jarett *et al*. [8] found that diets containing edible cricket did not negatively affect bacterial communities after feeding tropical house crickets to dogs.

Consumption of AD and/or BMp is widespread in United Kingdom, Spain, Brazil, Dominican Republic, Netherlands, Finland, Thailand, China, Indonesia, Vietnam, and South Korea [20, 21]. Moreover, these insects have the potential to be produced on a large scale. Therefore, edible insects could be an alternative protein source for dogs with many benefits, which pet owners should accept because many humans have also consumed these insect species.

Therefore, this study aimed to examine the fecal microbiota of dogs fed a diet that substituted poultry meal with AD or BMp at different levels.

## Materials and Methods

### Ethical approval

This study was approved by Institutional Animal Care and Use Committee, Kasetsart University, Bangkok, Thailand (Approval no. ACKU64-VET-010).

### Study period and location

The study was conducted in March 2021 at a designated experimental dog farm located in Nakhon Nayok, Thailand.

### Animals, diets, and experimental design

Fifteen healthy adult mixed-breed dogs (seven males and eight females) aged 3–5 years old, with an average weight of 22.5 ± 1.78 kg and a nine-scale body condition score of 4.13 ± 0.19 (mean ± standard error of mean), were randomly selected for the study from an experimental farm’s dog colony. No dogs had any gastrointestinal problems or taken supplements of prebiotics, probiotics, or antibiotics, and all of them passed a physical examination by a veterinarian with complete blood count and blood chemistry in the normal range. Each dog was housed in a separate pen in an open housing system throughout the experiment. This study was conducted at an experimental dog farm (Nakhon Nayok, Thailand).

Complete diets in semi-moist form were fed to the dogs for 29 days. The daily amount of feed was calculated based on the daily energy requirement recommendations of the Association of American Feed Control Officials (AAFCO) with a factor of 1.6 [22]. Fifteen dogs were systemically randomized and assigned to five groups consisting of three dogs each. These groups were fed a complete diet consisting of poultry meal (as a control group) or one with insect meal partially replacing poultry meal (10% or 20% AD or 7% or 14% BMp; as treatment groups). The nutrient chemical compositions of the experimental diet were analyzed [23] and are presented in Table-1 [22–24]. Dogs were fed once a day at 15:00 and provided with clean water *ad libitum*. The insects were purchased from a local company (Pathum Thani, Thailand). All insects were dried at 60°C for 48 h, ground to a size of 1 mm, and kept at −20°C until used to process the experimental diet. The experimental diets were formulated in isocaloric and isonitrogenic forms with nutritional profiles following the AAFCO [22] guidelines for maintaining an adult dog. The protein composition of insects was analyzed before formulating the experimental diet using a nitrogen-to-protein conversion factor of 4.76 [24], whereas 6.25 was used to determine crude protein in the experimental diets [23]. At the beginning of the experiment, the previous diets were switched to the experimental diet. Body weight, body condition score, and fecal samples were collected from each dog on 0, 14, and 29 days of the experiment. The fecal samples were stored under −20°C until analysis.

**Table-1:** Chemical composition of the diets for dogs during the experimental period.

Analyzed chemical composition (%DM)	Groups					AAFCO^1^

	Control	House cricket (*Acheta domesticus*)		Mulberry silkworm pupae (*Bombyx mori*)		
	
		10%	20%	7%	14%	
Moisture (%FM)	20.7	21.2	29.5	15.3	22.9	-
Calculated crude protein^2^	23.5	23.5	23.5	23.5	23.5	18.0
Analyzed crude protein^3^	25.3	26.4	29.2	25.0	25.7	18.0
Crude fat	10.5	10.3	10.2	10.0	10.4	5.50
Crude fiber	1.89	2.45	3.97	2.02	2.30	-
Ash	5.16	4.08	3.78	4.78	4.79	-
ME (kcal/kg in DM)^4^	3,776	3,785	3,737	3,763	3,772	-

1 Association of American Feed Control Officials 2021 dog food nutrient profiles based on dry matter recommendations for adult maintenance.

2 Nitrogen-to-protein conversion factors for analyzed composition were used at 4.76 [24] to reach isonitrogenic diet.

3 Nitrogen-to-protein conversion factors for analyzed composition were used at 6.25 [23].

4 Modified Atwater values [22]: Metabolizable energy or ME (kcal/kg) = (Protein×3.5) + (Fat×8.5) + (Carbohydrate × 3.5).

### Deoxyribonucleic acid (DNA) processing and 16S ribosomal ribonucleic acid (rRNA) gene sequencing

The microbial DNA extraction process was performed in accordance with the method of Sathitkowitchai *et al*. [25] using a bead meter and Qiagen QIAamp DNA stool kit (Qiagen, Hilden, Germany). Fecal samples were resuspended in phosphate-buffered saline (8 g of NaCl, 0.2 g of KCl, 1.44 g of Na_2_HPO_4_, and 0.24 g of KH_2_PO_4_) at pH 8 at a ratio of 1:4 w/v and pelleted as 1 mL of fecal slurry by centrifugation at 12,000× *g* for 2 min. The pellet was homogenized with 1 mL of lysis buffer and incubated in a heat block (Atuart Scientific, UK) at 70°C for 5 min. One milliliter of lysate was transferred to a 2 mL tube containing 0.3 g of each sterile zirconia bead with a diameter of 0.1 mm (BioSpec, Bartlesville, OK, USA). Mechanical lysis was conducted using a FastPrep-24 benchtop instrument (MP Biomedicals, Santa Ana, CA, USA) at 6.5 m/s 2 times with a series of 1 min beating and 5 min resting on ice. The supernatant was obtained after centrifugation at 12,000× *g* for 2 min, followed by the Qiagen QIAamp DNA stool kit protocol. Qualification and quantification of DNA were performed using a Nanodrop Spectrophotometer (Thermo Fisher Scientific, Waltham, MA, USA). Extracted DNA samples were immediately stored at −20°C. Paired-end reads were quality controlled and adapters were trimmed using fastp v0.21.0 [26] (read quality ≥q15 at 3’). The remaining high-quality paired-end reads were merged into single reads with FLASH [27] and the resulting reads were excluded if they were shorter than 210 bases. We processed these single reads into Amplicon Sequence Variances (ASVs) in R package DADA2 v.1.6 [28] with the default parameters. Taxonomy assignment was performed with QIIME2’s naïve Bayes classifier v2021.8 [29] using a 70% cutoff with the SILVA 138 99% OTU database [30]. ASVs that could not be identified at the phylum level and singleton ASVs were excluded from the analysis.

### Statistical analysis

**Figure-1:**
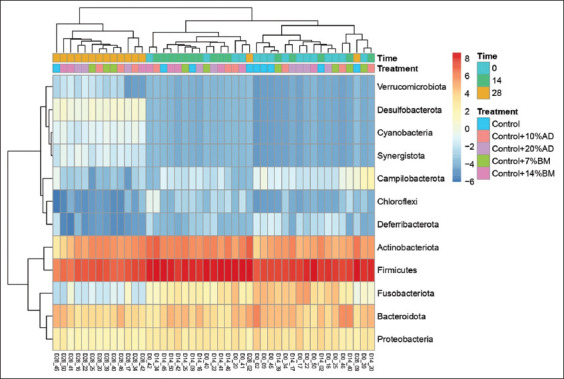
Heatmap diagram of the gut microbiota composition at phylum level of dogs fed poultry meal (control), 10 or 20% of house cricket (*Acheta domesticus*; AD), and 7 or 14% mulberry silkworm pupae (*Bombyx mori*; BMp) in different experimental period (Time). Cells are coloured by CLR-transformed of relative abundance of bacteria.

Diversity indices, including Chao1, Shannon, and Simpson were calculated using vegan package v2.5.6 (https://cran.r-project.org/) [31]. Distances between all samples were determined and ordination analysis was carried out using methods implemented in vegan package v2.5.6 [31]. A heatmap of bacterial abundance (Figure-1) was constructed using pheatmap R package v1.0.12 [32]. The principal coordinate analysis ordination with Bray–Curtis was calculated and visualized using ggplot2 R package v3.3.6. The differential relative abundance between groups was calculated using a two-way analysis of variance (ANOVA), in which group and time were used as independent variables. Duncan’s multiple range test was used as *post hoc* analysis after ANOVA. Both analyses were performed on the centered log-ratio transformation. In addition, analysis of compositions of microbiomes with bias correction was run as an additional differential abundance analysis test [33]. All statistical analysis and visualization were performed in R v4.1.2 [34]. Differences were accepted as being statistically significant at p < 0.05.

## Results

**Figure-2:**
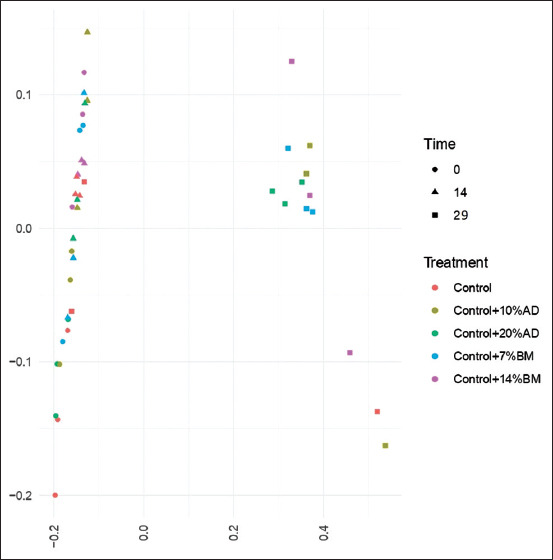
Principal coordinate analysis (PCoA) of 16S rDNA sequencing of the fecal microbiota in dogs fed poultry meal (control), 10 or 20% of house cricket (*Acheta domesticus*; AD), and 7 or 14% mulberry silkworm pupae (*Bombyx mori*; BMp) in different experimental period (Time).

On sequencing a total of 229,026 ± 2862 reads per sample and 1437 ASVs within the phylogenetic comparison based on a double hierarchical dendrogram, the five experimental groups were clustered together by time and treatment (Figure-1). The major phyla identified in this study were *Actinobacteria*, *Bacteroides*, *Firmicutes*, *Fusobacteria*, and *Proteobacteria*. In all samples, *Firmicutes*, *Actinobacteria*, and *Bacteroides* were most abundant. The results from the heatmap were similar to the beta diversity results (Figure-2). All experimental groups had a clearly unchanged bacterial community composition on days 0 and 14.

In contrast, differences were found in the dogs that had fed on the control diet for 29 days compared with the other groups. Only one dog in the control group was found to have a changed bacterial community composition, while the remaining two dogs in the control group had a bacterial community composition that was similar between days 0 and 14. In addition, the results of the cluster analysis of the treatment groups were unclear. The cluster analysis results for the treatment groups were inconclusive, as the heatmap did not provide a clear differentiation between each group in an overall assessment. To address this, a statistical analysis was conducted to elucidate variations in the relative abundance of individual bacterial species. The outcomes of this analysis are presented in Tables-2 and 3.

**Table-2:** Relative abundance (%) of major bacterial phyla in feces in dogs fed poultry meal or insect meals (Mean ± Standard error of mean).

Phylum	Groups					p-value

	Control	House cricket *(Acheta domesticus*)		Mulberry silkworm pupae (*Bombyx mori*)		
	
		10%	20%	7%	14%	
*Actinobacteria*	8.62 ± 1.63	11.8 ± 1.97	9.51 ± 1.52	12.1 ± 2.34	9.77 ± 1.72	0.548
*Bacteroides*	6.84 ± 3.00	4.06 ± 1.52	1.97 ± 0.36	3.32 ± 1.07	2.82 ± 1.13	0.389
*Firmicutes*	81.9 ± 2.55	83.0 ± 1.97	85.9 ± 1.26	83.3 ± 2.16	86.5 ± 1.81	0.509
*Fusobacteria*	1.56 ± 0.68	0.63 ± 0.34	1.90 ± 0.92	0.67 ± 0.37	0.27 ± 0.09	0.437
*Proteobacteria*	1.05 ± 0.35	0.41 ± 0.07	0.70 ± 0.20	0.53 ± 0.09	0.60 ± 0.16	0.216

**Table-3:** Relative abundance (%) of bacterial genera in feces in dogs fed poultry meal or insect meals (Mean ± Standard error of mean).

Genus	Groups					p-value

	Control	House cricket (*Acheta domesticus*)		Mulberry silkworm pupae (*Bombyx mori*)		
	
		10%	20%	7%	14%	
*Allobaculum*	14.2 ± 5.50^b^	13.7 ± 4.96^b^	11.6 ± 3.70^b^	11.1 ± 4.33^b^	3.44 ± 1.27^a^	0.040
*Bacteroides*	1.00 ± 0.42	0.74 ± 0.28	0.49 ± 0.09	0.71 ± 0.20	0.62 ± 0.34	0.570
*Bifidobacterium*	5.56 ± 1.71	7.42 ± 1.60	6.68 ± 1.33	8.01 ± 2.09	4.41 ± 0.91	0.479
*Blautia*	2.62 ± 0.61	2.16 ± 0.44	1.83 ± 0.27	2.50 ± 0.64	2.07 ± 0.48	0.745
*Catenibacterium*	2.61 ± 0.85	2.39 ± 1.05	0.93 ± 0.39	1.03 ± 0.17	1.37 ± 0.60	0.268
*Clostridium_sensu_stricto_1*	1.88 ± 0.37	1.25 ± 0.29	2.89 ± 1.01	1.22 ± 0.31	2.07 ± 0.63	0.517
*Collinsella*	1.58 ± 0.34	2.46 ± 0.59	1.56 ± 0.17	2.56 ± 0.51	1.94 ± 0.39	0.327
*Corynebacterium*	0.83 ± 0.27^a^	1.09 ± 0.38^a^	0.61 ± 0.14^a^	0.76 ± 0.12^a^	2.67 ± 0.91^b^	0.021
*Erysipelatoclostridium*	0.46 ± 0.12	0.59 ± 0.09	0.52 ± 0.07	0.42 ± 0.05	0.64 ± 0.12	0.210
*Faecalibacterium*	0.92 ± 0.37	1.06 ± 0.47	0.50 ± 0.11	0.47 ± 0.10	0.59 ± 0.27	0.260
*Fusobacterium*	1.51 ± 0.66	0.62 ± 0.34	1.65 ± 0.75	0.65 ± 0.36	0.26 ± 0.08	0.445
*Holdemanella*	3.04 ± 0.62	3.01 ± 0.72	1.64 ± 0.50	3.50 ± 0.93	3.56 ± 0.91	0.099
*Lactobacillus*	12.4 ± 2.58^a^	20.5 ± 4.70^a^	23.7 ± 4.12^ab^	23.1 ± 4.53^ab^	34.2 ± 2.89^b^	0.024
*Muribaculaceae*	3.20 ± 2.08	1.46 ± 1.18	0.71 ± 0.24	1.16 ± 0.80	0.36 ± 0.17	0.590
*Peptoclostridium*	7.96 ± 1.73	8.43 ± 1.77	7.00 ± 1.26	7.09 ± 0.76	6.93 ± 1.55	0.631
*Prevotella*	1.43 ± 1.18	0.59 ± 0.28	0.22 ± 0.08	0.84 ± 0.33	0.69 ± 0.42	0.732
*Romboutsia*	3.72 ± 0.63	2.40 ± 0.46	3.47 ± 0.68	2.15 ± 0.31	3.15 ± 0.78	0.250
*Ruminococcus_gnavus_group*	0.38 ± 0.07	0.61 ± 0.12	0.49 ± 0.06	0.58 ± 0.06	0.58 ± 0.10	0.229
*Streptococcus*	4.27 ± 1.32	2.83 ± 1.65	2.94 ± 1.02	6.25 ± 3.33	2.85 ± 0.93	0.217
*Turicibacter*	10.6 ± 2.51^b^	4.44 ± 0.96^a^	5.39 ± 1.28^ab^	6.14 ± 1.48^ab^	4.82 ± 1.49^a^	0.042

The difference on superscript letter in the same row represented the significant differences between groups (p < 0.05)

The results from measuring the alpha diversity of the dog fecal microbiota and firmicutes/bacteroides ratio (F/B ratio) in this study are shown in Table-4. There was no statistically significant difference in any diversity index or F/B ratio (p > 0.05) between the experimental groups. No interaction between group and date was presented on the alpha diversity index and F/B ratio, with the exception of Chao1. Chao1 remained steady during days 0–14 in all groups. However, a sharp increase in Chao1 was observed in all dogs fed insect meals on day 29, whereas the index in the control group remained steady as in the previous period.

**Table-4:** Alpha diversity measures of the fecal microbiota in dogs fed poultry meal or insect meals (Mean ± Standard error of mean).

Diversity index	Groups					p-value

	Control	House cricket (*Acheta domesticus*)		Mulberry silkworm pupae (*Bombyx mori*)		
	
		10%	20%	7%	14%	
Chao1	403 ± 48.4	492 ± 62.4	471 ± 57.3	493 ± 59.9	515 ± 66.6	0.540
Shannon	3.83 ± 0.16	3.77 ± 0.07	3.75 ± 0.07	3.72 ± 0.08	3.71 ± 0.14	0.961
Simpson	0.05 ± 0.01	0.06 ± 0.01	0.06 ± 0.01	0.07 ± 0.01	0.08 ± 0.01	0.458
F/Bratio^1^	53.3 ± 22.0	91.4 ± 44.2	58.5 ± 11.1	41.4 ± 9.82	124 ± 44.3	0.612

1 Firmicutes/Bacteroides ratio

The phylum level summary data for relative abundance are shown in Table-2. There was no statistically significant difference in relative abundance of the major bacterial phyla among the experimental groups fed poultry meal and insect meal (p > 0.05). Meanwhile, Table-3 illustrates the relative abundance at the genus level. With the diet containing 14% BMp, the level of *Allobaculum* was significantly lower and that of *Corynebacterium* was significantly higher compared with the findings in the other groups (p < 0.05). The levels of *Lactobacillus* in the control and 10% AD groups were markedly lower than those in the 14% BMp group (p < 0.05). The levels of *Turicibacter* were considerably lower in the 10% AD and 14% BMp groups than in the control group (p < 0.05).

## Discussion

The dog’s gut microbiome community relates to health and well-being and is affected by genetics, diets, physiological status, health, and environmental factors. The chemical composition of the diet is considered to be a major factor influencing it. However, major changes in the composition and proportion of the fecal microbiome hardly appear after using new ingredients with a different chemical composition to that of previous diets [8, 10, 11] or dietary supplementation [35] in healthy dogs. The same dogs fed prescription diets with four different levels of protein, fat, carbohydrate, and fiber (Satiety, Gastrointestinal low fat, renal, and Anallergenic prescription diets; Royal Canin) in different periods (4 × 4 Latin square design) did not show any modification in the dominant bacterial phyla [11]. In addition, replacing a chicken meal with tropical banded cricket powder (*G. sigillatus*) at a level of 24% of the formulation did not influence the dominant bacterial community [8]. As stated in the introduction, the consumption of tropical banded cricket powder by healthy humans did not lead to changes in bacterial diversity, and this aligns with the findings from the study in dogs [19]. In recent studies, dog diets were supplemented with several prebiotics, probiotics, and/or synbiotics to support gut health by creating a gut environment appropriate for beneficial bacteria [36]. As previously described by Beloshapka *et al*. [35], Gagné *et al*. [37] and Kim *et al*. [38], the diversity of the bacterial community did not change after supplementation with these biotics in healthy dogs. Only a few bacterial genera differed significantly in relative abundance between the experimental groups.

*Firmicutes* was found in the highest proportion of the fecal bacterial community in healthy dogs, followed by *Bacteroidetes*, *Fusobacteria*, *Proteobacteria*, and *Actinobacteria*, considered the major phyla [39]. The fecal microbiome of dogs fed a control diet containing cooked navy bean powder at 25% in the formulation did not change the bacterial diversity. However, this addition increased the number of *Firmicutes*, whereas *Actinobacteria* and *Fusobacteria* decreased based on the phylum level [10]. The cooked navy bean powder containing high levels of protein and fiber could cause these changes. Therefore, the high levels of AD and BMp used to replace poultry meal did not exert effects similar to the supplementation of a dogs’ diet with cooked navy bean powder. Based on this information, the integrity and stability of the gut bacterial community could be maintained in healthy dogs and were not disrupted by modifying the chemical composition of diets or supplementing them. In contrast, most of the bacterial community and diversity in dogs with inflammatory bowel disease were changed, with *Fusobacteria* being significantly decreased compared with the level in healthy dogs [40]. Therefore, the absence of a modification in the major bacterial community and diversity after being fed AD and BMp in this study is considered as the positive outcome that these proteins can be used in dogs without any adverse effects on the bacterial community.

However, a change in gut bacteria at the genus level was observed in this and other studies after feeding on different nutrient chemical compositions or supplementation. In contrast, the majority of the bacterial community and diversity did not change, as previously described by Jarett *et al*. [8], Kerr *et al*. [10], Mori *et al*. [11], and Stull *et al*. [19]. On feeding on weight-loss and low-fat diets, *Streptococcus* was significantly decreased, while *Faecalibacterium* was significantly increased, compared with the findings with an allergenic diet [11]. In this study, we formulated an isocaloric and isonitrogenic diet that considered the correction of the nitrogen-to-protein conversion factor in insect-based diets [24]. Furthermore, we ensured that the fat levels were nearly equal among the different diets. Therefore, the lack of differences observed may be attributed to the similarity in chemical composition between the diets, as reported in the study by Mori *et al*. [11]. The increases of *Catenibacterium*, *Lachnospiraceae* (*Ruminococcus*), and *Faecalitalea* and the decreases of *Bacteroides*, *Faecalibacterium*, and *Lachnospiraceae* NK4A136 group were reportedly observed in dogs fed diets with tropical banded cricket powder [8]. In contrast, an increase of *Bifidobacterium animalis* was demonstrated in humans who had consumed this cricket [19]. However, the levels of these bacteria were not changed between the control and insect dietary supplementation groups in this study. Therefore, the difference in insect species, formulation, dog colony, and chemical composition of the insects could be the causes of the different outcomes.

At the genus level, the amounts of *Allobaculum*, *Corynebacterium*, *Lactobacillus*, and *Turicibacter* were changed in the dogs fed 14% BM. In contrast, only a reduction in *Turicibacter* was found in the dogs fed 10% AD compared with the findings in the control group in this study. Therefore, the higher level of 14% BM had a minor influence on the bacterial community more than the AD and low-level BM supplementation at 7%. The majority of insects’ exoskeletons are chitin. Chitin and its derivatives are considered insoluble fibers with potential prebiotic properties to improve gastrointestinal health and change the gut microbiota [19]. The AD and BMp contained chitin at rates of 5.7% [41] and 18%, respectively [42]. Chitinase catalyzes the degradation of chitin to chitooligomers [43]. A previous study [44] showed that dog stomach, intestine, and colon tissue, including salivary tissue secrete Chia (acid chitinase) mRNA and its translation product to degrade a chitin substrate. However, dogs express a low-level of Chia compared with mice, chicken, and swine [44]. Previously, chitooligosaccharides were reported to enhance the growth of beneficial bacteria (*Lactobacillus rhamnosus*) and inhibit the growth of harmful bacteria such as *Escherichia coli* [45].

The presence of chitin in diets may be the reason for the elevation in *Lactobacillus* in the 14% BMp group. Further research could be conducted to test this hypothesis. In the present study, the high level of chitin in the insects fed to the dogs could have been the cause of the change in the minor microbiota in this study, compared with the low-level consumption of chitin that did not cause a change. Interestingly, the beneficial bacteria (*Lactobacillus* and *Corynebacterium*) in dogs in the 14% BM group increased in relative abundance. These results indicate that the ingestion of insects by dogs is a common event; therefore, alternative or novel protein sources in the pet food industry and chitin from insects will not cause an allergic reaction or health problems.

## Conclusion

This study was established to examine the fecal microbiota of dogs fed a diet that substituted common protein sources with AD or BMp at different levels. This study has shown that AD and BMp can substitute for common protein sources in canine diets with no statistically significant difference in any major gut microbiome phyla, diversity index, or F/B ratio (p > 0.05). Only a few bacterial genera significantly differed in their relative abundance between dogs fed a higher level of BMp at 14% of the diet (p < 0.05). Further studies should be conducted to assess the long-term effects of the stability and change of the gut microbiome and functional responses to health.

## Supplementary Material

Diet formulation (Table-S1) and Chao1 of the fecal microbiota in dogs fed poultry meal or insect meals (Mean ± Standard error of mean) in different periods (Table-S2).

## Authors’ Contributions

AK: Conceptualization. SA, AK, and PP: Methodology and data curation. PP, SA, AK and PC: Investigation and laboratory analysis. SA: Writing—original draft preparation. SA, PC, CL, PP, and AK: Writing—review and editing. AK: Project administration and funding acquisition. All authors have read, reviewed, and approved the final manuscript.

## Supplementary Tables

**Table-S1:** The experimental diets’ formulation.

Ingredients (%)	Groups				

	Control	House cricket (*Acheta domesticus*)		Mulberry silkworm pupae (*Bombyx mori*)	
	
		10%	20%	7%	14%
Corn	50.1	48.1	46.2	48.7	47.2
Soybean meal	10.0	10.0	10.0	10.0	10.0
Wheat flour	15.0	15.0	15.0	15.0	15.0
Poultry meal	17.4	10.0	2.51	13.3	9.10
House cricket meal	-	10.0	20.0	-	-
Mulberry silkworm pupae meal	-	-	-	7.00	14.0
Palm oil	5.72	5.09	4.44	4.01	2.50
Calcium carbonate	0.53	0.56	0.60	0.74	0.95
Salt	0.60	0.60	0.60	0.60	0.60
Vitamin premix^1^	0.15	0.15	0.15	0.15	0.15
Mineral premix^2^	0.20	0.20	0.20	0.20	0.20
Choline Chloride	0.30	0.30	0.30	0.30	0.30

1 Vitamin premix (Feed specialties Co., Ltd; Pathum Thani, Thailand) were supplied per kilogram of diets at 2,500,000 IU of vitamin A; 1,000,000 IU of vitamin D3; 7,000 IU of vitamin E; 700 mg of vitamin K; 400 mg of vitamin B1; 800 mg of vitamin B2; 400 mg of vitamin B6; 4 mg of vitamin B12; 30 mg of biotin; 3,111 mg of Ca pantothenate acid; 100 mg of folic acid; 15,000 mg of vitamin C; 5,600 mg of vitamin B3.

2 Mineral premix (Feed specialties Co., Ltd; Pathum Thani, Thailand) were supplied per kilogram of diets at 10,500 mg of Zn, 10,920 mg of Fe; 9,960 mg of Mn; 3,850 mg of Cu; 137 mg of I; 70 mg of Se

**Table-S2:** Chao1 of the fecal microbiota in dogs fed poultry meal or insect meals (Mean ± Standard error of mean) in different period.

Groups	Days			p-value		
	
	0	14	29	Groups	Days	Groups*Days
Control	368 ± 18.9	401 ± 8.15	439 ± 163	0.54	<0.001	0.008
House cricket (*Acheta domesticus*)						
10%	379 ± 13.7	356 ± 7.71	741 ± 2.56			
20%	340 ± 10.1	376 ± 16.5	697 ± 19.2			
Mulberry silkworm pupae (*Bombyx mori*)						
7%	374 ± 2.59	374 ± 14.2	731 ± 16.8			
14%	383 ± 0.69	386 ± 6.37	775 ± 46.0			
